# Genome Sequence of Coxsackievirus A6, Isolated during a Hand-Foot-and-Mouth Disease Outbreak in Finland in 2008

**DOI:** 10.1128/genomeA.01004-14

**Published:** 2014-10-16

**Authors:** Riikka Österback, Satu Koskinen, Pirjo Merilahti, Juha-Pekka Pursiheimo, Soile Blomqvist, Merja Roivainen, Asta Laiho, Petri Susi, Matti Waris

**Affiliations:** a Department of Virology, University of Turku, Turku, Finland; b National Institute for Health and Welfare (THL), Helsinki, Finland; c Turku Centre for Biotechnology, University of Turku and Åbo Akademi University, Turku, Finland; d Diagnostics and Industrial Microbiology Group, Turku University of Applied Sciences, Turku, Finland

## Abstract

Reports of hand-foot-and-mouth disease (HFMD) outbreaks caused by coxsackievirus A6 have increased worldwide after the report of the first outbreak in Finland in 2008. The complete genome of the first outbreak strain from a vesicle fluid specimen was determined.

## GENOME ANNOUNCEMENT

Coxsackievirus A6 (CV-A6) belongs to the *Enterovirus* A species within genus *Enterovirus*, in the *Picornaviridae* family. Before the hand-foot-and-mouth disease (HFMD) outbreak in Finland in 2008, only sporadic cases of CV-A6 had been reported and mainly with the symptom of herpangina (1). Thereafter, CV-A6 has been associated with HFMD outbreaks in several countries, with similar symptoms and homologous virus sequences (2–7). One main feature of HFMD caused by CV-A6 is onychomadesis, the nail shedding that occurs approximately 8 weeks after infection (2, 8). The RNA genome of CV-A6 is approximately 7,500 bases long, with noncoding regions in the 5′ (5′ NCR) and 3′ (3′ NCR) ends. The single open reading frame encodes a polyprotein consisting of three regions: P1, P2, and P3. P1 consists of the structural proteins VP1 to VP4. The nonstructural proteins of genome replication and protein synthesis are located in P2 and P3. The complete genome sequence of the prototype strain Gdula was the only full-length CV-A6 genome sequence determined before the outbreak in Finland in 2008, and currently, there are 16 CV-A6 genome sequences in GenBank (5, 9, 10). This study describes the genome sequence of a CV-A6 isolate from the Finnish outbreak in 2008.

The specimen was collected from an 8-year-old patient with HFMD symptoms of sore throat, high fever, and vesicles in the hands. CV-A6 was proliferated in RD cells (7) for up to two passages, followed by purification by sucrose density gradient centrifugation. Viral RNA was extracted from the purified virus preparation, and the virus identity was confirmed by a specific CV-A6 VP1 reverse transcription-PCR (RT-PCR) (2). The RNA was prepared for sequencing using the TruSeq RNA v2 sample preparation kit (Illumina) and sequenced on the Illumina MiSeq platform. CV-A6 prototype strain Gdula (accession no. AY421764) was used as the reference for genome mapping with the CLC Genomics Workbench version 5 analysis package (CLC bio). Identity calculations were performed with the PHYLIP software package.

The complete genome of the coxsackievirus strain A6/Finland/2008 (CV-A6^FI08^), constructed from 10^6^ paired reads with an average length of 146 nucleotides (nt), was 7,423 bp in length. Compared with the sequence of Gdula, the CV-A6^FI08^ sequence is 11 bp shorter in the 5′ NCR end and has a three-nucleotide insertion (ATT) at position 115 and a two-nucleotide deletion at position 7368 in the 3′ NCR. The nucleotide identities between Gdula and a CV-A6 strain from Taiwan (accession no. JQ946055) are 81% and 98%, respectively.

Similarity plots using the SimPlot program (11) revealed two major differences between CV-A6^FI08^ and CV-A6 Gdula compared to the traditional HFMD viruses CV-A16 (accession no. AF177911) and enterovirus A71 (EV-A71) (accession no. AF304457). The *cis*-acting replication element inside the 2A protease gene of CV-A6^FI08^, in contrast to Gdula, was highly similar to that of CV-A16 and EV-A71. In the 3A and 3D sites of the polyprotein, Asn^1467^ and Ser^2109^ in Gdula were replaced in CV-A6^FI08^ with Ser and Pro, respectively, as found also in CV-A16 and EV-A71.

It is tempting to think that some of the observed nucleotide and/or amino acid differences through their effects in replication cycles and the host process shutdown may explain the shift in symptoms from herpangina (Gdula) to more severe HFMD (CV-A6^FI08^).

### Nucleotide sequence accession number.

The complete genome sequence of coxsackievirus A6/Finland/2008 (CV-A6^FI08^) has been deposited in GenBank under the accession no. KM114057.
